# Supplementary material for: Comprehensive Multimorbidity Patterns in Older Patients Are Associated with Quality Indicators of Medication—MoPIM Cohort Study
Source: Int J Environ Res Public Health. 2022 Nov 29;19(23):15902. doi: 10.3390/ijerph192315902 (PMC9739326; doi:10.3390/ijerph192315902)
Supplement: Supplementary file 1 [file ijerph-19-15902-s001.zip › ijerph-2044846-supplementary.pdf]

International Journal of
*Environmental Research and Public Health*
MDPI

**Table S1.** Equivalences between names used in ADRs classification and ATC codes.

| Name | ATC Code |
|---|---|
| Analgesic | M01A |
| Angiotensin-converting enzyme (ACE) inhibitor | C09A |
| Angiotensin II receptor blocker | C09C |
| Antiarrhythmic | C01 |
| Antibiotic | J01 |
| Anticoagulant | B01AB |
| Antidepressant | N06A |
| Antiepileptic | N03A |
| Antiplatelet | B01AC |
| Antivitamin K | B01AA |
| Benzodiazepine | N05BA |
| Beta blocker | C07 |
| Bronchodilator | R03 |
| Corticoid | H02A |
| Loop diuretic | C03C |
| Hypnotic | N05C |
| Insulin | A10A |
| Neuroleptic | N05A |
| Opioid | N02A |
| Oral anticoagulant | B01AE, B01AF |
| Oral antidiabetic | A10B |
| Potassium sparing diuretic | C03D |
| Proton pump inhibitor | A02BC |
| Statin | C10AA |
| Thiazide diuretic | C03A |

ATC: Anatomical Therapeutic Chemical.

**Table S2.** Number and percentage of patients with each chronic condition or geriatric syndrome registered according to the assigned multimorbidity cluster.

| Chronic Condition or Geriatric Syndrome | Osteo-Articular | Psycho-Geriatric | Minor Chronic Disease | Cardio-Respiratory |
|---|---|---|---|---|
| Acute confusional syndrome/delirium | 51 (37.2) | 102 (67.5) | 29 (22.8) | 53 (16.3) |
| Amputation | 7 (5.1) | 2 (1.3) | 4 (3.1) | 3 (0.9) |
| Anaemia | 77 (56.2) | 64 (42.4) | 76 (59.8) | 117 (36.0) |
| Asthma | 32 (23.4) | 11 (7.3) | 10 (7.9) | 29 (8.9) |
| Cardiac arrhythmia | 86 (62.8) | 77 (51.0) | 77 (60.6) | 183 (56.3) |
| Cerebrovascular disease | 40 (29.2) | 53 (35.1) | 36 (28.3) | 59 (18.2) |
| Chronic gastritis or gastro-oesophageal reflux | 25 (18.2) | 11 (7.3) | 19 (15.0) | 39 (12.0) |
| Chronic pain | 127 (92.7) | 82 (54.3) | 88 (69.3) | 105 (32.3) |
| Chronic renal insufficiency | 81 (59.1) | 50 (33.1) | 73 (57.5) | 116 (35.7) |
| Chronic thyroid disease | 44 (32.1) | 19 (12.6) | 23 (18.1) | 49 (15.1) |
| Cognitive/Intellectual impairment | 32 (23.4) | 130 (86.1) | 35 (27.6) | 32 (9.8) |
| Constipation | 78 (56.9) | 88 (58.3) | 41 (32.3) | 133 (40.9) |
| COPD | 41 (29.9) | 38 (25.2) | 49 (38.6) | 145 (44.6) |
| Degenerative arthropathy | 111 (81.0) | 79 (52.3) | 81 (63.8) | 114 (35.1) |
| Dementia | 17 (12.4) | 98 (64.9) | 24 (18.9) | 40 (12.3) |

| | | | | |
|---|---|---|---|---|
| Depression or anxiety | 84 (61.3) | 52 (34.4) | 37 (29.1) | 95 (29.2) |
| Diabetes mellitus with organ damage | 28 (20.4) | 21 (13.9) | 34 (26.8) | 50 (15.4) |
| Diabetes mellitus without organ damage | 27 (19.7) | 57 (37.7) | 36 (28.3) | 81 (24.9) |
| Drug-related conditions | 22 (16.1) | 19 (12.6) | 7 (5.5) | 19 (5.8) |
| Dyslipidaemia | 89 (65.0) | 50 (33.1) | 88 (69.3) | 133 (40.9) |
| Dysphagia | 36 (26.3) | 78 (51.7) | 28 (22.0) | 17 (5.2) |
| Frailty | 115 (83.9) | 138 (91.4) | 59 (46.5) | 145 (44.6) |
| Gallstones (previous hepatic colic) | 18 (13.1) | 12 (7.9) | 16 (12.6) | 33 (10.2) |
| Gout | 30 (21.9) | 14 (9.3) | 54 (42.5) | 39 (12.0) |
| Heart failure | 89 (65.0) | 79 (52.3) | 71 (55.9) | 204 (62.8) |
| Hematologic disorders | 8 (5.8) | 3 (2.0) | 14 (11.0) | 11 (3.4) |
| Hypertension | 121 (88.3) | 119 (78.8) | 111 (87.4) | 252 (77.5) |
| Immobility | 101 (73.7) | 143 (94.7) | 55 (43.3) | 131 (40.3) |
| Incontinence (urinary/fecal) | 33 (24.1) | 112 (74.2) | 16 (12.6) | 31 (9.5) |
| Inflammatory osteoarticular disease | 19 (13.9) | 4 (2.6) | 12 (9.4) | 14 (4.3) |
| Instability/falls | 93 (67.9) | 59 (39.1) | 51 (40.2) | 83 (25.5) |
| Ischemic heart disease without infarction | 38 (27.7) | 17 (11.3) | 27 (21.3) | 38 (11.7) |
| Malnutrition | 12 (8.8) | 65 (43.0) | 5 (3.9) | 65 (20.0) |
| Mild liver disease | 5 (3.6) | 7 (4.6) | 7 (5.5) | 13 (4.0) |
| Moderate or severe liver disease | 3 (2.2) | 3 (2.0) | 1 (0.8) | 12 (3.7) |
| Myocardial infarction | 28 (20.4) | 11 (7.3) | 26 (20.5) | 46 (14.2) |
| Neoplasia | 16 (11.7) | 18 (11.9) | 25 (19.7) | 52 (16.0) |
| Neurologic disorder of the central nervous system | 11 (8.0) | 9 (6.0) | 4 (3.1) | 8 (2.5) |
| Non-ischemic heart disease | 58 (42.3) | 31 (20.5) | 58 (45.7) | 91 (28.0) |
| Obesity | 58 (42.3) | 24 (15.9) | 43 (33.9) | 68 (20.9) |
| Osteoporosis | 49 (35.8) | 13 (8.6) | 12 (9.4) | 29 (8.9) |
| Parkinson's disease | 10 (7.3) | 9 (6.0) | 5 (3.9) | 10 (3.1) |
| Peptic ulcer disease | 10 (7.3) | 8 (5.3) | 12 (9.4) | 16 (4.9) |
| Peripheral neuropathy or neuritis | 21 (15.3) | 9 (6.0) | 18 (14.2) | 13 (4.0) |
| Peripheral vascular disease | 21 (15.3) | 13 (8.6) | 22 (17.3) | 49 (15.1) |
| Polypharmacy | 133 (97.1) | 115 (76.2) | 123 (96.9) | 220 (67.7) |
| Pressure ulcers | 10 (7.3) | 60 (39.7) | 10 (7.9) | 15 (4.6) |
| Previous fractures (not hip) | 57 (41.6) | 28 (18.5) | 19 (15.0) | 35 (10.8) |
| Previous hip fracture | 22 (16.1) | 14 (9.3) | 4 (3.1) | 27 (8.3) |
| Rheumatologic disease | 8 (5.8) | 3 (2.0) | 5 (3.9) | 16 (4.9) |
| Sensory deficit | 58 (42.3) | 70 (46.4) | 49 (38.6) | 137 (42.2) |
| Sleep apnoea | 29 (21.2) | 7 (4.6) | 18 (14.2) | 12 (3.7) |
| Sleep disorders/Insomnia | 90 (65.7) | 86 (57.0) | 51 (40.2) | 106 (32.6) |
| Varicose veins | 59 (43.1) | 25 (16.6) | 43 (33.9) | 37 (11.4) |
| Vertigo | 42 (30.7) | 13 (8.6) | 7 (5.5) | 15 (4.6) |

COPD: chronic obstructive pulmonary disease.

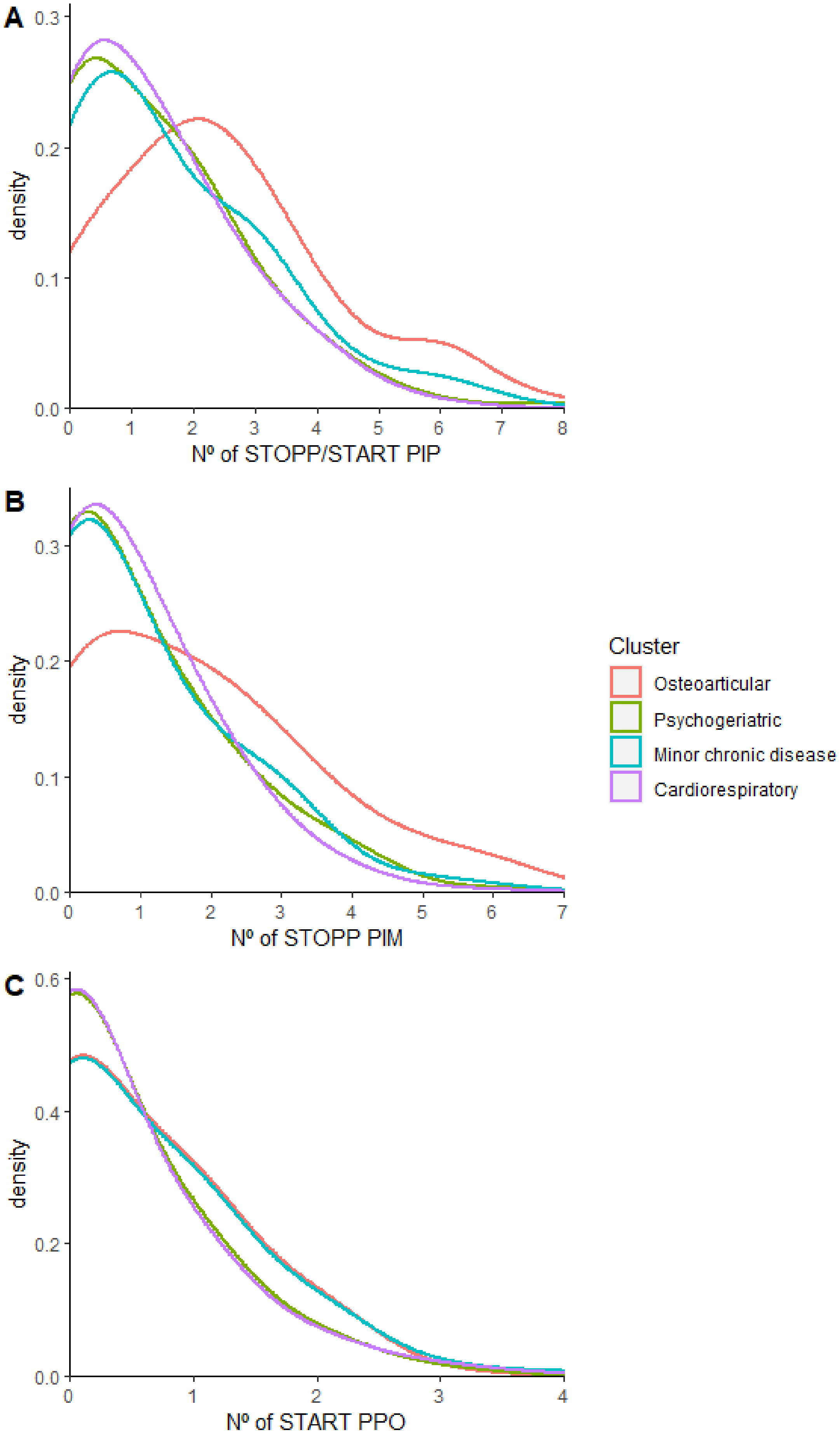
A
density
0.3
0.2
0.1
0.0
0
1
2
3
4
5
6
7
8
Nº of STOPP/START PIP
B
density
0.3
0.2
0.1
0.0
0
1
2
3
4
5
6
7
Nº of STOPP PIM
C
density
0.6
0.4
0.2
0.0
0
1
2
3
4
Nº of START PPO
Cluster
Osteoarticular
Psychogeriatric
Minor chronic disease
Cardiorespiratory

**Figure S1. A**: Distribution of the number of STOPP/START PIP according to the assigned multimorbidity cluster. Pairwise comparisons between cluster distributions performed with the Kolmogorov-Smirnov test showed significant differences between the osteoarticular cluster and the rest ($p < 0.001$). PIP: Potentially inappropriate prescribing. **B**: Distribution of the number of STOPP PIM according to the assigned multimorbidity cluster. Pairwise comparisons between cluster distributions performed with the Kolmogorov-Smirnov test showed significant differences between the osteoarticular cluster and the rest ($p < 0.005$). PIM: potentially inappropriate medication. **C**: Distribution of the number of START PPO according to the assigned multimorbidity cluster. Pairwise comparisons between cluster distributions performed with the Kolmogorov-Smirnov test showed no significant differences ($p > 0.05$). PPO: potential prescribing omission.

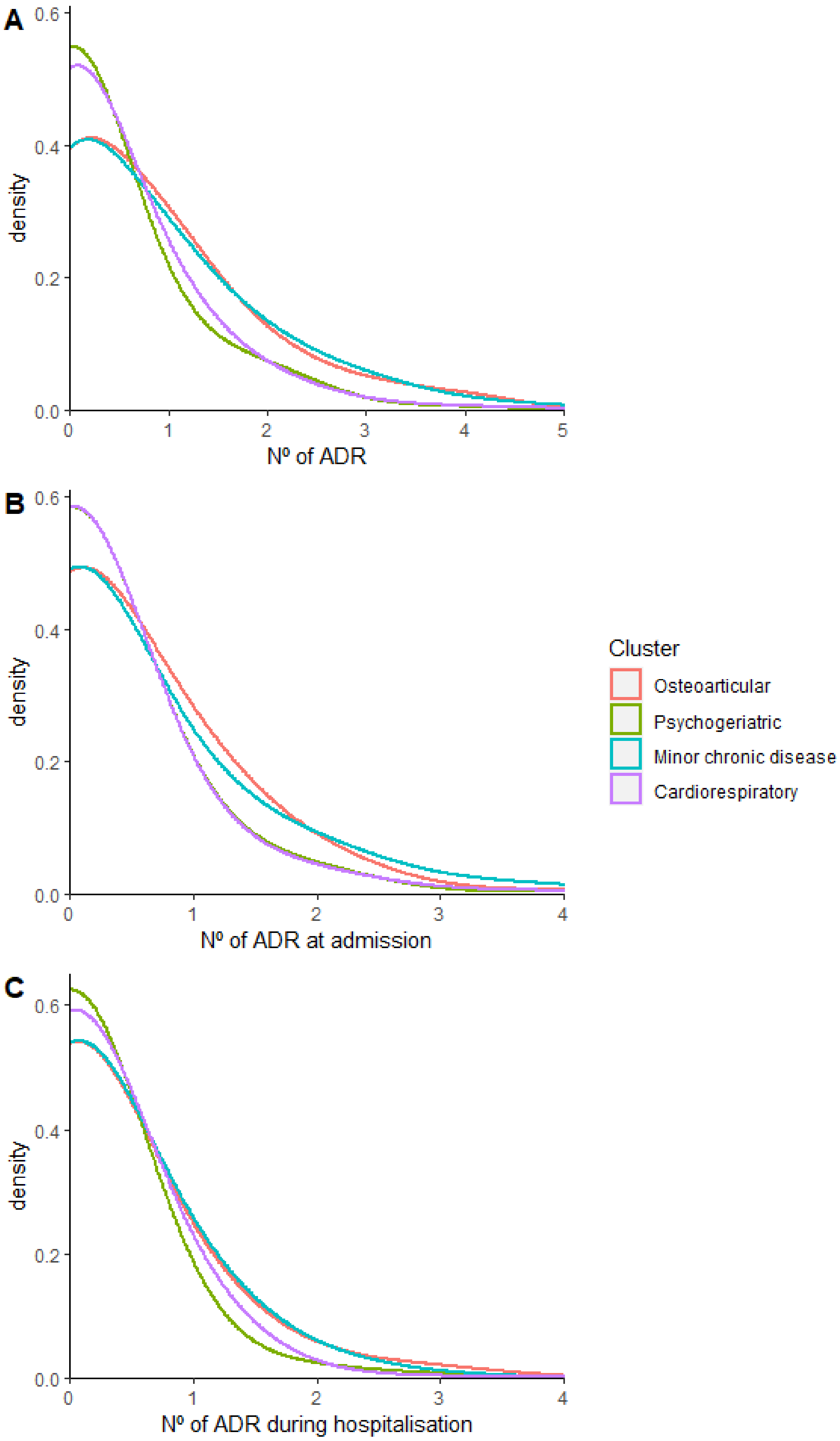
A
0.6
0.4
0.2
0.0
density
0
1
2
3
4
5
Nº of ADR
B
0.6
0.4
0.2
0.0
density
0
1
2
3
4
Nº of ADR at admission
C
0.6
0.4
0.2
0.0
density
0
1
2
3
4
Nº of ADR during hospitalisation
Cluster
Osteoarticular
Psychogeriatric
Minor chronic disease
Cardiorespiratory

**Figure S2. A**: Distribution of the number of ADRs according to the assigned multimorbidity cluster. Pairwise comparisons between cluster distributions performed with the Kolmogorov-Smirnov test showed significant differences between the osteoarticular and the minor chronic disease clusters and the rest ($p < 0.001$). **B**: Distribution of the number of ADRs at admission according to the assigned multimorbidity cluster. Pairwise comparisons between cluster distributions performed with the Kolmogorov-Smirnov test showed significant differences between the osteoarticular cluster and the minor chronic disease clusters and the rest ($p < 0.05$). **C**: Distribution of the number of ADRs during admission according to the assigned multimorbidity cluster. Pairwise comparisons between cluster distributions performed with the Kolmogorov-Smirnov test showed no significant differences ($p > 0.05$). ADR: adverse drug reaction.

**Table S3.** *p*-values of post-hoc pairwise.

| | Osteoarticular vs. Psychogeriatric | Osteoarticular vs. Minor Chronic Disease | Osteoarticular vs. Cardiorespiratory | Psychogeriatric vs. Minor Chronic Disease | Psychogeriatric vs. Cardiorespiratory | Cardiorespiratory vs. Minor Chronic Disease |
|---|---|---|---|---|---|---|
| Any PIP | **<0.001** | **0.009** | **<0.001** | 0.347 | 0.815 | 0.19 |
| Any PIM | **0.014** | **0.014** | **0.002** | 1 | 0.779 | 0.779 |
| Any PPO | **0.037** | 1 | **0.012** | **0.037** | 0.998 | **0.012** |

Fisher's exact test assessing for association between the presence of any potentially inappropriate prescribing (PIP), potentially inappropriate medication (PIM) or potential prescribing omission (PPO), according to STOPP/START criteria, and multimorbidity cluster belonging (osteoarticular, psychogeriatric, minor chronic disease or cardiorespiratory). Benjamini-Hochberg correction was applied at a false discovery rate of 5%. $p < 0.05$ are shown in bold.

**Table S4.** *p*-values of post-hoc pairwise.

| | Osteoarticular vs. Psychogeriatric | Osteoarticular vs. Minor Chronic Disease | Osteoarticular vs. Cardiorespiratory | Psychogeriatric vs. Minor Chronic Disease | Psychogeriatric vs. Cardiorespiratory | Cardiorespiratory vs. Minor Chronic Disease |
|---|---|---|---|---|---|---|
| Any STOPP A1: Acetylsalicylic acid | 0.103 | **0.044** | 0.102 | 0.602 | 1 | 0.287 |
| Any STOPP A1: Proton pump inhibitor | 0.519 | 0.519 | **0.001** | 0.213 | **<0.001** | **0.022** |
| Any STOPP A2 | 0.641 | 0.643 | 0.198 | 0.266 | 0.643 | 0.06 |
| Any STOPP B11 | **0.013** | 0.112 | **<0.001** | 0.444 | 0.311 | 0.055 |
| Any STOPP D5 | **<0.001** | **0.003** | **0.003** | 0.568 | 0.192 | 0.568 |
| Any STOPP G5 | **0.004** | 0.088 | **0.007** | 0.4 | 0.436 | 0.823 |
| Any STOPP I1 | 0.571 | 0.061 | 1 | **0.023** | 0.469 | **0.042** |
| Any STOPP K1 | **<0.001** | **<0.001** | **<0.001** | 0.866 | 0.086 | 0.146 |
| Any STOPP K2 | 0.103 | 0.771 | **0.003** | **0.04** | **<0.001** | **0.015** |
| Any STOPP L2 | **0.047** | 0.356 | **0.047** | 0.522 | 1 | 0.522 |

Fisher's exact test assessing for association between the presence of any STOPP criteria previously significantly associated, and multimorbidity cluster belonging (osteoarticular, psychogeriatric, minor chronic disease or cardiorespiratory). Benjamini-Hochberg correction was applied at a false discovery rate of 5%. $p < 0.05$ are shown in bold.

**Table S5.** *p*-values of post-hoc pairwise.

| | Osteoarticular vs. Psychogeriatric | Osteoarticular vs. Minor | Osteoarticular vs. Cardiorespiratory | Psychogeriatric vs. Minor | Psychogeriatric vs. Cardiorespiratory | Cardiorespiratory vs. Minor Chronic Disease |
|---|---|---|---|---|---|---|

| | | Chronic Disease | | Chronic Disease | | |
|---|---|---|---|---|---|---|
| Any START A8 | 0.854 | 0.854 | **0.037** | 1 | **0.01** | **0.01** |
| Any START C2 | 0.112 | 1 | 0.06 | 0.144 | 1 | 0.062 |
| Any START C3 | 0.138 | 0.165 | 1 | 0.877 | 0.082 | 0.138 |
| Any START E2 | 0.169 | 0.427 | **0.02** | 0.594 | 0.594 | 0.384 |
| Any START E5 | 0.506 | 0.506 | **0.003** | 0.185 | **0.037** | **<0.001** |
| Any START H2 | **0.007** | 0.68 | **0.007** | **0.039** | 0.802 | **0.039** |

Fisher's exact test assessing for association between the presence of any START criteria previously significantly associated, and multimorbidity cluster belonging (osteoarticular, psychogeriatric, minor chronic disease or cardiorespiratory). Benjamini-Hochberg correction was applied at a false discovery rate of 5%. $p < 0.05$ are shown in bold.

**Table S6.** *p*-values of post-hoc pairwise.

| | Osteoarticular vs. Psychogeriatric | Osteoarticular vs. Minor Chronic Disease | Osteoarticular vs. Cardiorespiratory | Psychogeriatric vs. Minor Chronic Disease | Psychogeriatric vs. Cardiorespiratory | Cardiorespiratory vs. Minor Chronic Disease |
|---|---|---|---|---|---|---|
| Any ADR | **<0.001** | 0.902 | **<0.001** | **<0.001** | 0.168 | **<0.001** |
| Any ADR at admission | **0.001** | 0.95 | **<0.001** | **0.002** | 1 | **<0.001** |
| Any ADR during admission | **0.001** | 1 | **0.041** | **0.001** | **0.041** | **0.041** |

Fisher's exact test assessing for association between the presence of any adverse drug reaction (ADR), detected at the time of admission or during admission, and multimorbidity cluster belonging (osteoarticular, psychogeriatric, minor chronic disease or cardiorespiratory). Benjamini-Hochberg correction was applied at a false discovery rate of 5%. $p < 0.05$ are shown in bold.

**Table S7.** *p*-values of post-hoc pairwise.

| | Osteoarticular vs. Psychogeriatric | Osteoarticular vs. Minor Chronic Disease | Osteoarticular vs. Cardiorespiratory | Psychogeriatric vs. Minor Chronic Disease | Psychogeriatric vs. Cardiorespiratory | Cardiorespiratory vs. Minor Chronic Disease |
|---|---|---|---|---|---|---|
| Any ADR ACE inhibitor | **0.093** | 0.169 | **0.01** | 0.788 | 0.727 | 0.442 |
| Any ADR Neuroleptic | 0.371 | 0.732 | 1 | 0.472 | 0.087 | 0.472 |
| Any ADR ARB | 0.059 | 0.609 | 0.099 | **0.038** | 0.528 | **0.045** |
| Any ADR Insulin | 0.727 | 0.106 | 0.727 | **0.039** | 1 | **0.005** |
| Any ADR Loop diuretic | 0.094 | 0.446 | 0.145 | 0.296 | 0.446 | 0.446 |
| Any ADR Thiazide diuretic | 0.412 | 0.412 | 0.54 | 0.083 | 0.54 | 0.083 |

Fisher's exact test assessing for association between the presence of any adverse drug reaction (ADR) previously significantly associated, and multimorbidity cluster belonging (osteoarticular, psychogeriatric, minor chronic disease or cardiorespiratory). Benjamini-Hochberg correction was applied at a false discovery rate of 5%. $p < 0.05$ are shown in bold. ACE: angiotensin-converting enzyme. ARB: angiotensin II receptor blocker.
